# Diversity of Volatile Profiles and Nutritional Traits Among 29 Cucumber Cultivars

**DOI:** 10.3390/foods14223878

**Published:** 2025-11-13

**Authors:** Panling Lu, Chunfang Wang, Yongxue Zhang, Haijun Jin, Shaofang Wu, Xiaotao Ding, Hongmei Zhang

**Affiliations:** 1 Shanghai Key Laboratory of Protected Horticultural Technology, Horticulture Research Institute, Shanghai Academy of Agricultural Sciences, 1000 Jinqi Road, Fengxian District, Shanghai 201403, China; lpl2245@163.com (P.L.); zhangyongxue@saas.sh.cn (Y.Z.); jinhaijun@saas.sh.cn (H.J.); sfwu@saas.sh.cn (S.W.); 2 Crop Breeding & Cultivation Research Institute, Shanghai Academy of Agricultural Sciences, 1000 Jinqi Road, Fengxian District, Shanghai 201403, China; fhwcf@126.com

**Keywords:** cucumber, volatile compounds, Fruit quality, cultivars, HS-SPME-GC-MS

## Abstract

Twenty-nine samples of two cucumber types (*Cucumis sativus* L.) were evaluated to determine the amino acid, tannin, soluble protein, soluble sugar, Vc, nitrate nitrogen contents and volatile organic compounds (VOCs). Cucumber variety differences in amino acid, tannin, soluble proteins, et al., were significant (*p* < 0.05). The VOCs were derived by headspace solid-phase microextraction (HS-SPME) coupled with gas chromatography–mass spectrometry (GC-MS). A total of 67 VOCs were identified, including 24 aldehydes, 13 alcohols, 12 ketones, 12 alkenes and 6 other compounds. (E, Z)-2,6-Nonadienal, (E)-2-Nonenal and (E)-6-Nonenal were the three most abundant volatiles. A total of 21 VOCs were present in all 29 cultivars. An average of 45 kinds of VOCs were identified in each cultivar. Principal component analysis (PCA) clustered the 29 cucumber cultivars into five groups. Partial least-squares-discriminant analysis (PLS-DA) indicated that the European type was separated from the South China type across PLS1. Furthermore, 25 key differential volatiles for distinguishing 29 cultivars and 23 key differential volatiles for differentiating between South China and European types were identified, respectively. These results provide information for the development of new cultivars with high nutritional quality and intense flavor.

## 1. Introduction

Cucumber (*Cucumis sativus* L.), a member of the Cucurbitaceae family, is a globally cultivated and consumed vegetable [1]. Cucumber is very popular because of its distinctive flavor and nutritional value, which are rich in beneficial nutrients, including vitamin C, sugars, proteins, amino acids, flavonoids, and polyphenolic compounds, which contribute to human health [2]. Aroma, a crucial component of fruit flavor, is closely linked to volatile organic compounds (VOCs), and is vital for fruit quality assessment and breeding [3,4]. In recent years, with increasing consumer demand for better flavor in cucumbers, growing research is being conducted on cucumber volatile compounds [5,6]. A comprehensive analysis of the volatile compounds in cucumbers will not only provide a theoretical foundation for flavor quality improvement but also offer guidance for optimizing postharvest preservation techniques and processing technologies.

Since the 1960s, there has been significant attention on the research and regulation of flavor compounds in cucumbers [7,8]. The aromatic compounds in cucumbers are primarily composed of volatile organic substances such as aldehydes, alcohols, ketones, and esters [9]. Among them, aldehydes are the main flavor compounds in cucumbers, accounting for 56.30–83.92% of the total volatile substances, followed by alcohols [10]. Studies have shown that C6 and C9 volatile compounds are the main components of cucumber aroma [2]. C6 volatile compounds impart the characteristic fresh green and grassy notes, while C9 aldehyde has a floral aroma [11], the ratio of these two groups of components plays a decisive role in the flavor of cucumbers [12]. It has been determined based on flavor threshold and content that (E, Z)-2,6-Nonadienal and (E)-2-Nonenal are the primary aroma-active compound in cucumber fruit, playing a dominant role in its characteristic flavor [13,14,15]. Plant VOCs can be roughly divided into three main categories based on their synthesis pathways: the terpenoids pathway, amino acid pathway and fatty acids pathway [5,6,12]. The main aromatic compounds in cucumbers are aldehydes and alcohols, primarily produced through the fatty acid metabolic pathway. The unique aroma of cucumber is primarily derived from linoleic acid and linolenic acid, which are oxygenated under the catalysis of LOX (lipoxygenase), introducing molecular oxygen at the C13 or C9 position to form 13-hydroperoxy linolenic acid (13-HPOT) or 9-hydroperoxy linolenic acid (9-HPOT). These compounds are then converted by hydroperoxide lyase (HPL) into short-chain aldehydes and oxoacids, ultimately producing the pleasant, fresh cucumber fragrance [16,17].

Aroma is strongly dependent on genotypes. Long-term selection and breeding practices have led to the development of distinct cucumber ecotypes and market classes, such as Chinese Long (North or South China type, Japanese Long), European Long (English or Dutch cucumber), and North American slicing or pickling cucumbers [18]. In studies on cucumber fruit flavor, significant differences have been observed in flavor intensity among different cucumber genotypes. Zhang et al. analyzed the aroma compounds of three cucumber types (European greenhouse, Northern Chinese-type, and Southern Chinese-type) and found that different varieties of cucumber contain different types and amounts of VOCs [5]. Aroma is also closely related to their varietal characteristics, ripeness, and postharvest handling [17,19,20].

Various methods are available for determining volatile organic compounds (VOCs), such as dynamic headspace extraction [21], stir bar sorptive technique [22,23], and solid-phase microextraction (SPME) [24]. Solid-phase microextraction (SPME), as a simple and solvent-free technique for volatile compound extraction, has been widely used in conjunction with gas chromatography–mass spectrometry (GC-MS) for the qualitative and quantitative analysis of volatile compounds in fruits and vegetables [25,26].

Optimizing flavor is one of the primary objectives in breeding superior cucumber varieties and developing processed products. Therefore, it is essential to investigate volatile flavor compounds across different cucumber genotypes to further understand and exploit genetic resources. Current research on cucumber fruit aroma compounds has primarily focused on the identification of flavor substances within the fruit [27], the dynamic changes in aromatic compounds during different developmental stages [17], and the key factors influencing fruit flavor quality [15]. While existing studies have characterized volatile profiles in various cucumber cultivars, our understanding of the organic volatile composition in diverse breeding materials remains incomplete. Research on cucumber volatiles has largely been limited to a few cultivars, and studies systematically comparing different cucumber types are lacking. In this study, headspace solid-phase microextraction (HS-SPME) coupled with gas chromatography–mass spectrometry (GC-MS) was used to analyze the composition and concentration of volatile compounds in 29 cucumber cultivars (16 European types and 13 South China types). The objectives were to (1) assess aroma quality at the cultivar level, (2) evaluate inter-varietal differences, thereby providing a scientific basis for future cultivation and breeding strategies, and (3) identify the essential differences in volatile compounds between European and South China cucumber types, providing the chemical rationale for their unique flavor characteristics. These findings offer critical data for mining, utilizing, and enhancing proprietary, high-quality cucumber germplasm.

## 2. Materials and Methods

### 2.1. Plant Materials

**Figure 1:**
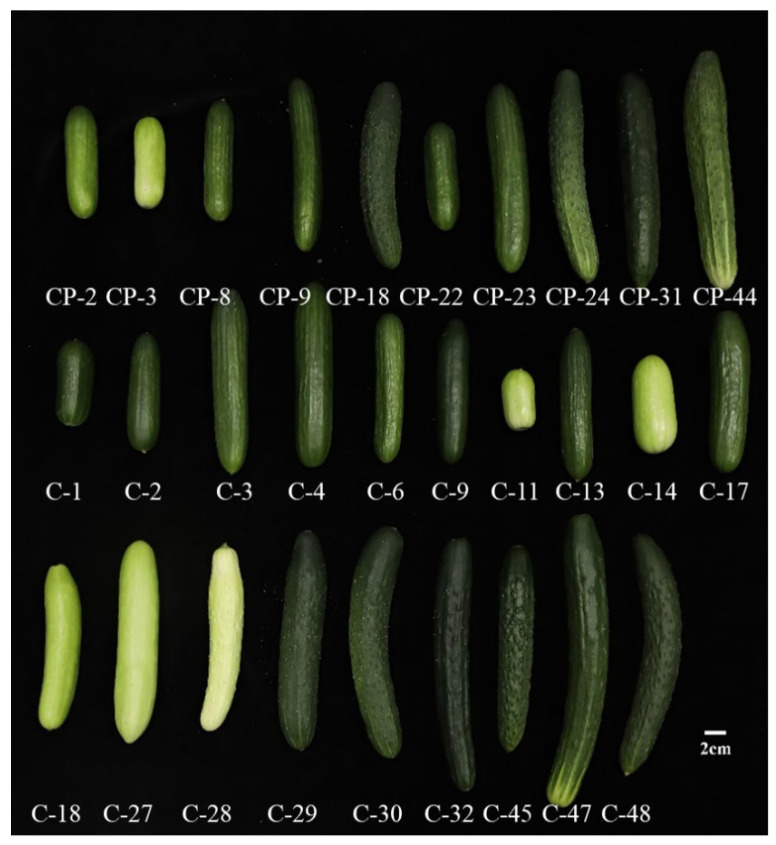
Materials of 29 cucumber cultivars used in this study.

In this study, 29 Cucumber cultivars (19 inbreds and 10 F1 hybrids) were grown in the plastic greenhouse at Zhuanghang Comprehensive Experimental Station, Shanghai Academy of Agricultural Sciences, China. All cucumber cultivars were grown in a single greenhouse under standardized conditions, with soil, water, and fertilizer management maintained constant. Among them, C-1 to C-4, C-6, C-9, C-11, C-13, C-14, C-17, C-18, C-27 to C 30, C-32, C-45, C-47, and C-48 were inbreds, and CP-2, CP-3, CP-8, CP-9, CP-18, CP-22, CP-23, CP-24, CP-31, and CP-44 were hybrids. More details and pictures of cucumber fruits are listed in Figure 1 and Table S1. The fruits were harvested at 10–12 days after flowering. Nine fruits for each cultivar were harvested, each replicate containing 3 fruits. The cucumber fruit was divided into three segments based on length, and then intermediate segments were chopped into small pieces and immediately frozen in liquid nitrogen, and stored at −80 °C until analysis.

### 2.2. Physiological Characteristics Measurement

The single fruit weight was measured by an electronic balance, fruit length and flesh thickness were determined with a straight ruler, and fruit diameter was measured by a vernier caliper. Total soluble solids (TSS) content was measured using a digital refractometer. The amino acid (ninhydrin method), tannin (Folin–Ciocalteu method), soluble protein (Coomassie Brilliant Blue method), soluble sugar (anthrone–sulfuric acid method), Vc (2,6-dichlorophenol-indophenol titration), and nitrate nitrogen contents (salicylic acid colorimetric method) were determined using assay kits (Suzhoukeming, Suzhou, China), following the manufacturer’s protocols.

### 2.3. HS-SPME Analysis

Before analysis, the samples were ground by a cryogenic grinding machine (JX-FSTPRP-I, Shanghai Jingxin Industrial Development Co., Ltd., Shanghai, China). The HS-SPME analysis was performed following the study described by Wei et al. [28], and the appropriate adjustments were made. For the extraction of volatile compounds, 7 g of the ground sample was added into a 20 mL vial and 1 g of NaCl and 8 µL 2-octanol solution (100 μg/L, added as internal standard) were also added. All the extractions were performed using SPME fiber composed of 50/30 μm carboxen divinylbenzene polydimethylsiloxane (CAR/DVB/PDMS) (Supelco Inc., Bellefonte, PA, USA). The extraction fiber was heated and aged for 5 min in the aging device in advance. Samples were equilibrated at 40 °C for 15 min with agitation. The fiber was inserted into the headspace of the capped vial to adsorb volatile substances for 30 min. After extraction, the fiber underwent thermal desorption in the GC inlet at 260 °C for 5.0 min.

### 2.4. GC–MS Analysis

GC-MS conditions were according to the procedure described by Xu et al. [29]. A GC–MS system (7890B-5977B, Agilent Technologies Inc., Palo Alto, CA, USA) equipped with a DB-WAX capillary column (30 m × 250 μm × 0.25 μm) was used for analysis. The GC temperature was programmed as follows: 40 °C held for 5 min, raised to 220 °C at 5 °C/min, then increased at 20 °C/min to 250 °C and held for 2.5 min. Helium, the carrier gas, was circulated at 1.0 mL/min at a constant flow rate in splitless mode. The temperature of the ion source was 230 °C and transfer line was 260 °C. Blank runs were conducted during sample analyses. MS fragmentation was performed under an electron ionization of 70 eV with the scan range of 29–450 *m*/*z*.

### 2.5. Identification and Quantification of Volatile Compounds

MassHunter software (B.07.05.2479) was used to collect the data from the GC-MS; every composition was analyzed by the computer workstation’s automatic deconvolution system (AMDIS) and volatile compounds were identified by the database of NIST/EPA/NIH Mass Spectral Library (NIST 2014) according to its mass fragmentation pattern from the spectra database. Only substances with an MS matching score greater than 70% were retained. The concentration of each compound in cucumber was calculated using the internal standard method with reference to Wei’s method. The calculation formula is as follows: C_x_ (μg/Kg) = S_x_ × C_i_/S_i_/1000. C_x_ represents the concentration of the aroma substance; S_x_ represents the peak area of the aroma substance; C_i_ represents the concentration of internal standard 2-octanol; S_i_ represents the peak area of internal standard 2-octanol.

### 2.6. Statistical Analysis

All the data were the means of three biological replicates. Excel 2021 software was used for statistical analysis and charting of data. SPSS 22.0 software was employed for analyzing data using Duncan’s multiple range tests of variance (*p* < 0.05) and significance test. The heatmap of the major volatiles was produced by TBtools-II, and the PCA and PLS-DA were produced by the online website of MetaboAnalyst 6.0 (https://www.metaboanalyst.ca).

## 3. Results and Discussion

### 3.1. Comparison of Nutrient Contents

The levels of Vc, amino acids, soluble solids, soluble sugars, soluble proteins, tannins, and nitrites in cucumbers are important indicators for evaluating their nutritional quality. The soluble solids content directly affects the nutritional quality of cucumber fruits. Meanwhile, the contents of Vc, soluble sugars, and soluble protein indirectly influence the nutritional quality of the fruits [30]. Enhancing the content of beneficial components such as Vc, amino acids, and soluble sugars, while reducing the levels of tannins and nitrites, is a primary focus in cucumber quality breeding and high-quality cultivation practices [31]. There was diversity in these nutrient contents among 29 cucumber cultivars (Table 1). The Vc and amino acids contents varied from 31.14 to 182.57 mg/kg FW and 224.54 to 782.02 mg/kg FW, with a mean of 82.32 mg/kg FW and 422.68 mg/kg FW. The soluble proteins, soluble sugars, and SSC ranged from 0.34 to 2.22 mg/g FW, 11.51 to 29.06 mg/g FW, and 3.6 and 5.37, with a mean of 0.98 mg/g FW, 19.37 mg/g FW and 4.26, respectively. The tannins and nitrate nitrogen ranged from 38.39 to 98.14 mg/kg FW and 41.97 to 237.13 mg/kg FW; the average values were 69.98 mg/kg FW and 112.92 mg/kg FW, respectively. These values are consistent with the results reported in other studies [31,32,33].

### 3.2. Identification of Volatile Compounds

Cucumber fruits are deeply loved by people for their unique aroma, and flavor compounds are an important indicator for evaluating fruit quality [5]. With the advancement of detection technologies, an increasing number of aromatic substances in cucumbers have been identified. GEORGIOS et al. employed capillary gas chromatography–mass spectrometry (GC/MS) to analyze the volatile components of three Greek cucumber varieties, identifying a total of 21 aroma compounds [34]. Min et al. identified 84 VOCs in 69 cucumber genotypes, including 15 unknown compounds [35]. In this study, 67 volatile compounds were detected, including 24 aldehydes, 13 alcohols, 12 ketones, 12 alkenes, and 6 other compounds (Table 2). An average of 45 kinds of VOCs were identified in each cultivar. CP-44 had the most VOCs (51), while C-4 and C-14 had the fewest VOCs (38). More than 50 types of VOCs were identified in C-27 (50). Fewer than 45 types of VOCs were identified in C-2 (43), C-4 (38), C-6 (40), C-14 (38), CP-2 (40), CP-8 (44), CP-22 (42), and CP-23 (43) (Table 3); 21 VOCs were present in all cultivars, including Hexanal, (E)-2-Hexenal, 2-Octanone, Nonanal, (E, Z)-2,6-Nonadienal, (E)-2-Nonenal, et al. (Table S2).

The formation of cucumber aroma is attributed to a combination of various volatile compounds, with the composition and concentration of these aromatic substances demonstrating distinct patterns across different varieties [9]. In this study, the VOCs of the 29 varieties showed significant differences, ranging from 771.34 ± 66.75 µg/kg FW to 1802.38 ± 53.11 µg/kg FW (Table 3). C-9 had the highest content of VOCs, followed by C-28 (1630.5 ± 188.25 µg/kg FW) and C-18 (1571.32 ± 78.57 µg/kg FW), while CP-31 (771.34 ± 66.75 µg/kg FW) had the lowest content. C-9 were 2.3-fold greater than those in CP-31. An analysis of total volatile compounds in 15 F1 cucumber hybrids derived from 4 maternal and 10 paternal parents by Min et al. revealed that the total volatile content in the F1 generation was lower than the mid-parent value, particularly in terms of ethanol content [35]. In this study, CP-31 (a hybrid cultivar) exhibited the lowest volatile content, while C-9 (an inbred line) showed the highest volatile content, which is consistent with the aforementioned research findings.

### 3.3. Composition and Concentration of Volatile Compounds

**Figure 2:**
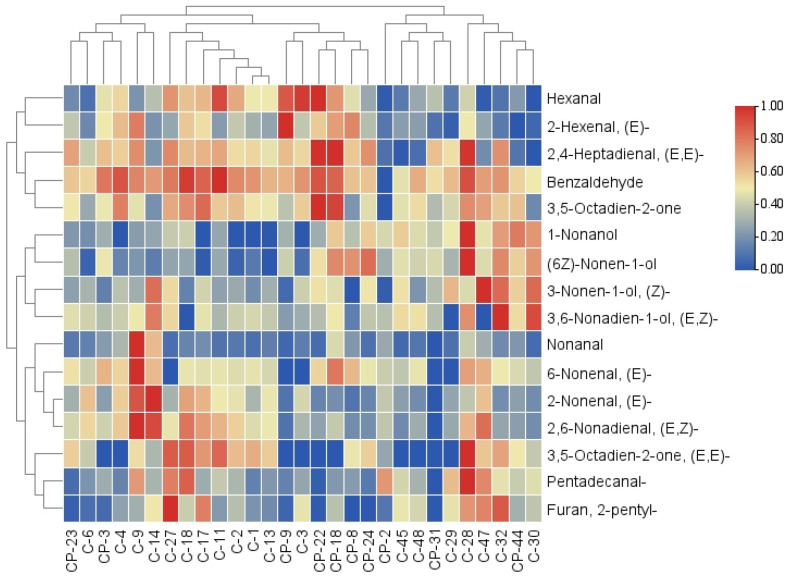
Contents (as shown by heatmap) and hierarchical cluster analysis of the major volatiles (average content > 15 µg/kg FW) in the fruits of 29 cucumber cultivars.

Aldehydes, alcohols, ketones, alkenes, and other VOCs were identified in 29 cucumber cultivars; the composition and concentration of VOCs are shown in Table S3. For all cultivars, the main types were aldehydes and alcohols (Table 4), which contributed to 80–90% of the total volatile content (Table S4), similar to previous studies [15]. While the consensus is that aldehydes and alcohols are the main substances responsible for the formation of cucumber flavor, this does not mean that other volatile compounds can be overlooked [14]. This is because aroma depends not only on the concentration of volatiles but also on their thresholds [36]. Among these VOCs, major volatiles (16 types, average content > 15 µg/kg FW) are presented in Figure 2. As shown in Table 2, (E, Z)-2,6-Nonadienal (A16), (E)-2-Nonenal (A15), (E)-6-Nonena (A12), Nonanal (A9), (E, E)-2,4-Heptadienal (A13), Hexanal (A4), Pentadecanal (A22), and 3,5-Octadien-2-one (K6) were the most abundant volatiles (average content > 50 μg/kg FW) in 29 cucumber cultivars.

#### 3.3.1. Aldehydes

Aldehydes were the most abundant volatiles in cucumber; among all cultivars, twenty-four aldehydes were identified (Table 2), accounting for between 65.71% (C-32) and 85.83% (C-6) (Table S4); the average value was 76.64%, which is similar to previous studies [10,15]. The content of aldehydes varies significantly among different cucumber cultivars; the highest total aldehyde content was observed in C-9 (1531.18 ± 36.56 µg/kg FW), accounting for 84.95% of the total VOCs, while the lowest was found in CP-31 (548.39 ± 52.23 µg/kg FW), making up 71.10% of the total VOCs (Table S4). Among the aldehydes, (E, Z)-2,6-Nonadienal (A16) was the most abundant (163.06 ± 13.57 to 479.77 ± 5.25 µg/kg FW), followed by (E)-2-Nonenal (A15) (93.36 ± 10.27 to 380.42 ± 36.91 µg/kg FW) and (E)-6-Nonenal (A12) (0 to 184.01 ± 5.67 µg/kg FW) (Table S3). A total of nine aldehydes had average concentrations exceeding 15 µg/kg FW including the three mentioned above, as well as Hexanal (A4), (E)-2-Hexenal (A8), Nonanal (A9), (E, E)-2,4-Heptadienal (A13), Benzaldehyde (A14), and Pentadecanal (A22) (Table 2 and Table S3). Among these aldehydes, eleven compounds were identified in all cultivars (Table S2). (E, Z)-2,6-Nonadienal is primarily responsible for cucumber’s flavor, imparting a fresh cucumber scent, and (E)-2-Nonenal is the second most important odor compound in cucumbers [37]. These two compounds have low odor thresholds and significantly influence the flavor of cucumber fruits [38]. In this study, C-9 had a higher content of (E, Z)-2,6-Nonadienal than other cucumber cultivars, up to 479.77 ± 5.24 µg/kg FW. C-14 had a higher content of (E)-2-Nonenal, up to 380.42 ± 36.91 µg/kg FW. Previous studies have indicated that a higher ratio of (E, Z)-2,6-Nonadienal to (E)-2-Nonenal correlates with a more intense fresh cucumber-like flavor [17]. The highest and lowest ratios were found in CP-24 and C-17, respectively. The average ratio is slightly smaller in European-type cucumbers compared to the South China type, but the difference is not significant. Hao et al.’s study demonstrated that (E)-6-Nonenal, Hexanal, (E)-2-Hexenal, Nonanal, and (E,E)-2,4-Heptadienal are also characteristic aroma compounds in cucumber, based on odor activity value (OAV) calculations [10,17]. The flavor of cucumbers is closely related to their genotypes. JO et al. found that the contents of most aldehydes, including 2,6-Nonadienal, Propanal, Pentanal, 2,4-Nonadienal, 2-Octenal, Tetradecanal, Hexadecanal, Hexanal, and 2-Hexenal, were relatively lower in the Korean group of cucumbers compared to other types such as Japanese, Chinese, and European cucumbers [9]. In our study, we did not find a similar pattern, which is likely attributable to the large sample size of the same cucumber type.

#### 3.3.2. Alcohols

In this study, a total of thirteen alcohols were detected, accounting for 4.87% (C-17) to 18.23% (C-30) of the total VOCs, with an average contribution of 9.84% (Table S4). Alcohol content varied considerably among different cultivars. The highest total alcohol content was observed in C-32 (202.17 ± 16.08 µg/kg FW, 15.62% of total VOCs), while the lowest was in C-13 (68.68 ± 3.7 µg/kg FW, 5.86% of total VOCs). 1-Nonanol (L7), (Z)-3-Nonen-1-ol (L8), (6Z)-Nonen-1-ol (L9), and (E, Z)-3,6-Nonadien-1-ol (L10) were the main alcohol compounds (Table 2, >15 µg/kg). Among these, (6Z)-Nonen-1-ol and (Z)-3-Nonen-1-ol exhibited the highest concentrations, at 26.48 µg/kg FW (ranging from 8.42 ± 1.3 to 55.49 ± 7.37) and 25.84 µg/kg FW (ranging from 17.55 ± 5.89 to 38.05 ± 2.21), respectively. Four alcohols were detected in all cultivars, including Ethanol (L1), 1-Hexanol (L2), (Z)-3-Nonen-1-ol (L8), and (6Z)-Nonen-1-ol (L9) (Table 2 and Table S3). Previous studies indicate that (6Z)-Nonen-1-ol and (E, Z)-3,6-Nonadien-1-ol contribute floral notes, and (E)-2-hexene-1-ol and (E)-3-hexene-1-ol possess a distinctive grassy aroma [17]. Therefore, alcohols also have a large influence on cucumber flavor.

#### 3.3.3. Ketones

Ketones have a floral and fruity sweet flavor [39]. Twelve ketones were identified, contributing between 4.27% (C-14) and 10.95% (C-32) of the total VOCs, with an average of 8.42% (Table S4). C-32 had the highest content (141.72 ± 9.64 µg/kg FW, 10.95% of total volatiles) of all ketones, followed by C-27 (139.89 ± 8.48 µg/kg FW, 10.79% of total volatiles) and C-17 (139.37 ± 7.73 µg/kg FW, 9.46% of total volatiles), whereas C-14 had the lowest total ketones (66.81 ± 1.79 µg/kg FW, 4.27% of total volatiles) (Table S4). 3,5-Octadien-2-one and (E, E)-3,5-Octadien-2-one were the main ketones (Table 2, >15 µg/kg), accounting for 69.30% of all ketones. 2-Octanone, 3,5-Octadien-2-one, and trans-beta-Ionone were the three ketones present in all cucumber cultivars.

#### 3.3.4. Alkenes

A total of 12 alkenes compounds were detected in 29 cucumber fruits, accounting for 0.07% (C-6) to 4.68% (C-18) of the total VOCs, with an average of 2.04% (Table S4). C-18 had the highest total olefins (73.59 ± 4.37 µg/kg FW, 4.68% of total volatiles), while C-6 had the lowest total alkenes (0.83 ± 0.07 µg/kg FW, 0.07% of total volatiles). The principal alkenes were Caryophyllene and Humulene; however, they were not identified in all cucumber cultivars, and no alkenes present in all cucumber cultivars.

#### 3.3.5. Others

One ester and five furans were found, constituting 0.78% to 6.94% of the total volatile substances (Table S4). Esters constitute the main body of aromatic compounds and impart the characteristic aroma to various fruits and vegetables such as apples and melons [40,41]. Furan is an oxygen-containing heterocyclic compound that imparts fruity and fresh aromas [42]. 2-pentyl-Furan (F2) had the highest content, ranging from 2.06 ± 0.23 µg/kg in cultivar CP-8 to 41.29 ± 11.43 µg/kg in cultivar C-27 (Table 2 and Table S3). 2-ethyl-Furan(F1) and 2-pentyl-Furan (F2) were detected in all cultivars. It has been reported that the furan compound contents in cucumbers of the North China type and South China type are higher than in the European type [43], but no similar pattern was observed in this study.

### 3.4. Analysis of Volatile Compounds

**Figure 3:**
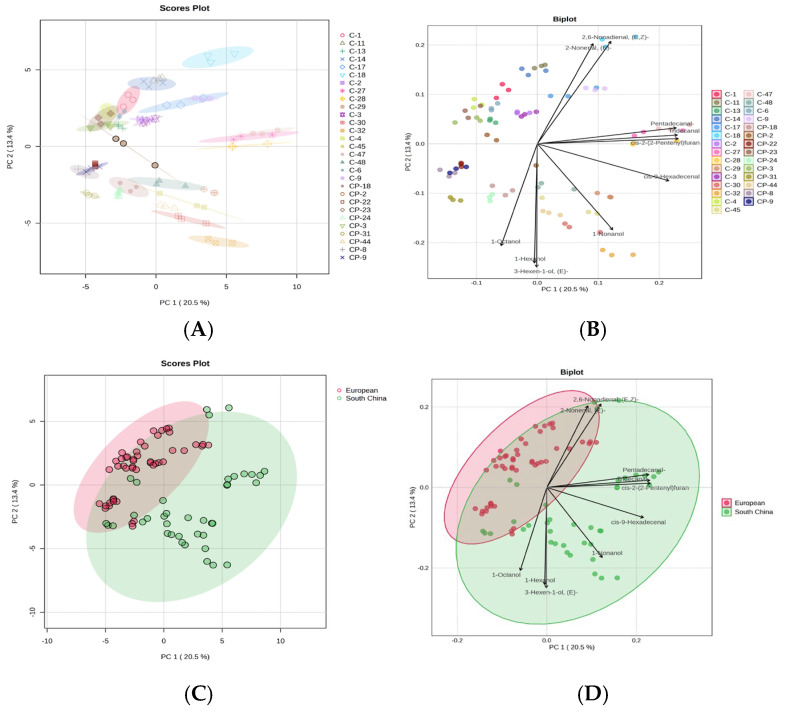
PCA score plots of 29 cucumber cultivars based on all VOC data. (**A**,**C**) shows the PCA scores scatter plot. (**B**,**D**) shows the PCA loading plots.

Principal component analysis (PCA) is an unsupervised clustering method, often used to provide a partial visualization of data in dimensionality reduction plots, and it has been widely applied in the study of volatile components [44,45]. Principal component analysis (PCA) was performed to confirm the differences in 67 VOCs among 29 breeding lines cultivar. Overall, 67 VOCs could be simplified into four principal components (PCs), with a cumulative variance contribution rate of 53.7%. The first two principal components (PCs) accounted for 33.9%, PC1 and PC2 represented 20.5% and13.4% of the total variance, respectively (Figure 3A). The 29 cultivars were divided into five groups based on the relationship between scores and loadings (Figure 3B). The first group included three cultivars (C-27, C-28, C-47), which contained high relative contents of Pentadecanal (A22), Tridecanal (A19), cis-9-Hexadecenal (A24), and cis-2-(2-Pentenyl) furan (E4). The second group contained one cultivar (C-18) characterized by high relative contents of (E, Z)-2,6-Nonadienal (A16) and (E)-2-Nonenal (A15). The third group contained twelve cultivars (C-1, C-2, C-3, C-4, C-9, C-11, C-13, C-14, C-17, CP-2, CP-3, CP-23) characterized by high relative contents of (+)-epi-Bicyclosesquiphellandrene (O12). The fourth group contained four cultivars (CP-8, CP-9, CP-22, CP-31) characterized by high relative contents of 2-Pentenal, 2-methyl- (A6), and 2-Butenal (A3). The fifth group contained eight cultivars (C-29, C-30, C-32, C-48, C-45, CP-18, CP-24, CP-44) characterized by high relative contents of 1-Octanol (L6), 1-Nonanol (L7), 1-Hexanol (L2), and 3-Hexen-1-ol, (E)- (L3). It is worth noting that the cultivars of the European type and South China type exhibit distinct separation, although a small number of varieties still show overlap (Figure 3C). The cultivars of the South China type were characterized by high relative contents of Pentadecanal (A22), Tridecanal (A19), cis-9-Hexadecenal (A24) and cis-2-(2-Pentenyl) furan (E4), 1-Octanol (L6), 1-Nonanol (L7), 1-Hexanol (L2) and 3-Hexen-1-ol, and (E)- (L3) (Figure 3D). Varieties within the same group do not necessarily belong to the same ecotype; the third and fourth groups contain both European-type and South China-type. A similar result was also observed by Shi et al. [26] and Min et al. [35].

**Figure 4:**
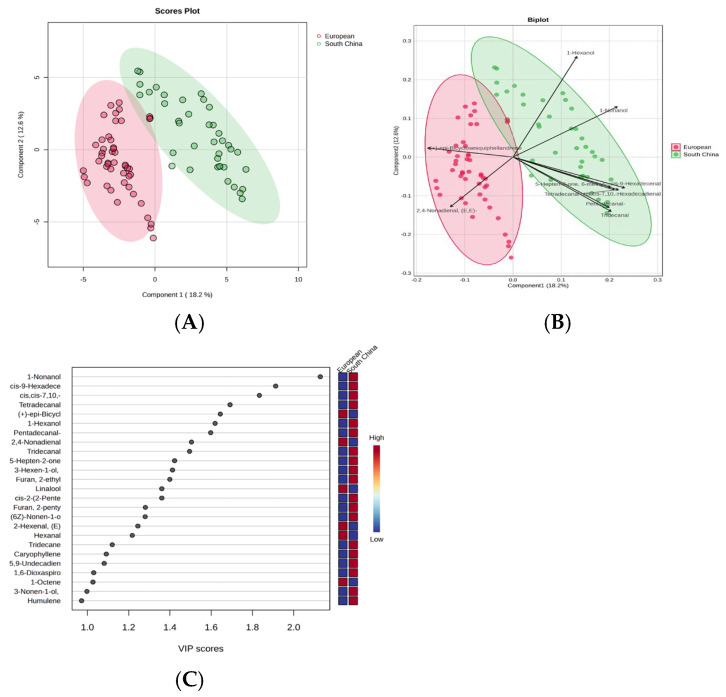
PLS-DA score plots of 29 cucumber cultivars of two types based on all VOC data. (**A**) shows the PLS-DA scores scatter plot. (**B**) shows the PLS-DA loading plots. (**C**) VOCs with VIP values larger than 1.0.

Partial least-squares-discriminant analysis (PLS-DA), a supervised classification technique, is widely applied to create multivariate models that predict features, differentiate samples, and screen for key biomarker variables [46]. Therefore, PLS-DA was performed to elucidate the differences among 29 cultivars in more detail. The results of the permutation test (R2 = 0.717, Q2 = 0.681) and (R2 = 0.833, Q2 = 0.783) demonstrated that both models exhibited favorable fitting without the risk of overfitting. Q2 > 0.5 is generally considered to indicate that the model has good predictive ability [47]. Previous studies show that PLS-DA model analysis could cluster cucumbers together depending on the geographical group to which they belong [9]. In this study, PLS-DA models from VOC analysis revealed that the cucumber fruit clustered together depending on the types. The European type was separated from the South China type across PLS1 (18.2%) (Figure 4A). The result of the loading plot was consistent with the PCA loading plot. The cultivars of South China-types have high relative contents of Pentadecanal (A22), Tridecanal (A19), et al., while European types have high relative contents of 2,4-Nonadienal, (E,E)- (A18) and (+)-epi-Bicyclosesquiphellandrene (O12) (Figure 4B). In the PLS-DA model, the VIP (Variable Importance in Projection) score estimates the importance of each x-variable in the predictive model for each x-variable and summarizes the contribution of variables to the model [48]. Typically, a VIP value greater than 1 is considered the criterion for variable selection [49]. The VIP plot of 29 cultivars indicates that 25 compounds with a VIP value larger than 1.0 were selected as the most discriminant VOCs, including 6 aldehydes, 7 alcohols, 3 ketones, 3 alkenes, and 6 others (Table S5). The VIP identified 23 compounds as key differential VOCs in different types of cucumbers (Table S6; Figure 4C), including 1-Nonanol, cis-9-Hexadecenal, Pentadecanal, et al. The content of 1-Nonanol, (E,E)-2,4-Nonadienal, Linalool, Hexanal, Tridecane, and 1-Octenewere higher in European type, while the content of 1-Nonanol, cis-9-Hexadecenal, cis,cis-7,10,-Hexadecadienal, Tetradecanal, et al. were higher in the South China type. Jo et al. [9]. conducted a comparative analysis of cucumber varieties from Korea, Europe, and Thailand, 22 and 17 significantly discriminant metabolites in the peel and pulp were identified by PLS-DA analysis, respectively. Some of these differential metabolites are consistent with the findings of this study, such as (E,E)-2,4-Nonadienal, Hexanal, and others.

Through PCA and PLS-DA analysis, potential volatile substances in different varieties can be purposefully identified, thereby uncovering differential information among different varieties. This will contribute to a comprehensive evaluation of cucumber volatile substances.

## 4. Conclusions

In this study, the amino acids, tannins, soluble proteins, soluble sugars, Vc, nitrate nitrogen contents, and compositions and content of VOCs in 29 cucumber cultivars were identified. As a result, cucumber variety differences in amino acids, tannins, soluble proteins, et al. were found to be significant (*p* < 0.05). A total of 67 VOCs were identified, including 24 aldehydes, 13 alcohols, 12 ketones, 12 alkenes, and 6 other compounds; 21 VOCs were common in all 29 cultivars, including Hexanal, (E)-2-Hexenal, 2-Octanone, Nonanal, (E, Z)-2,6-Nonadienal, (E)-2-Nonenal, et al. The content and variety of aroma substances differ significantly among different cultivars. An average of 45 kinds of VOCs were identified in each cultivar. CP-44 had the most kinds of VOCs, while C-4 and C-14 had the fewest kinds of VOCs. C-9 had the highest content of VOCs, while CP-31 had the lowest content. PCA clustered the 29 cucumber cultivars into five groups based on the relationship between scores and loadings. At the same time, the cultivars of the European and South China types exhibited distinct separation by PCA and PLS-DA analysis. The cultivars of the South China types showed high relative contents of Pentadecanal (A22), Tridecanal (A19) et al., while European-types had high relative contents of (E,E)-2,4-Nonadienal (A18) and (+)-epi-Bicyclosesquiphellandrene (O12). PLS-DA analysis also identified 25 key differential volatiles for distinguishing 29 cultivars and 23 key differential volatiles for differentiating between South China and European types. These results provide effective information for breeding distinct cucumber varieties, such as intensely aromatic types with high soluble sugar content suitable for fresh consumption, and types with stable flavor profiles and high solid content ideal for processing, thereby catering to diverse consumer preferences.

## Figures and Tables

**Table 1:** Comparison of nutrient contents in 29 cucumber cultivars.

Name	Amino Acid (mg/kg)	Tannin (mg/kg)	Soluble Protein (mg/g)	SSC (°Brix)	Soluble Sugar (mg/g)	Vc (mg/kg)	Nitrate Nitrogen (mg/kg)
C-1	593.26 ± 8.39 ^bcd^	53.77 ± 4.74 ^lm^	0.86 ± 0.06 ^fghi^	4.57 ± 0.15 ^bcd^	25.21 ± 2.32 ^bc^	71.48 ± 0.98 ^ijkl^	95.68 ± 3.29 ^gh^
C-2	527.80 ± 8.00 ^bcde^	54.34 ± 1.35 ^klm^	0.78 ± 0.03 ^hi^	4.3 ± 0.1 ^def^	25.89 ± 1.78 ^ab^	69.36 ± 2.22 ^jkl^	123.75 ± 3.92 ^de^
C-3	782.02 ± 30.45 ^a^	76.82 ± 4.37 ^defg^	0.84 ± 0.04 g^hi^	5.17 ± 0.15 ^a^	18.34 ± 1.11 ^hijkl^	76.48 ± 4.66 ^hijk^	97.19 ± 1.03 ^gh^
C-4	625.58 ± 33.06 ^b^	93.82 ± 3.67 ^ab^	0.84 ± 0.03 g^hi^	3.97 ± 0.06 ^fghijk^	22.63 ± 0.83 ^bcdef^	85.68 ± 2.84 ^ghi^	92.10 ± 4.29 ^ghi^
C-6	609.39 ± 47.84 ^bc^	62.52 ± 5.13 ^hijkl^	0.86 ± 0.08 fg^hi^	4.37 ± 0.06 ^cde^	23.28 ± 0.49 ^bcdef^	80.04 ± 2.40 ^hijk^	93.76 ± 6.92 ^gh^
C-9	487.10 ± 47.52 ^ef^	79.84 ± 6.38 ^cdef^	0.72 ± 0.02 ^hij^	3.97 ± 0.06 ^fghijk^	21.77 ± 0.15 ^cdefg^	95.53 ± 6.20 ^fg^	41.97 ± 3.92 ^l^
C-11	609.43 ± 63.54 ^bc^	98.14 ± 4.02 ^a^	1.22 ± 0.02 ^bcd^	4.77 ± 0.06 ^b^	22.65 ± 0.91 ^bcdef^	69.43 ± 5.91 ^jkl^	145.80 ± 2.50 ^cd^
C-13	518.46 ± 2.02 ^cde^	57.08 ± 3.73 ^klm^	0.54 ± 0.09 ^ijk^	3.93 ± 0.06 ^fghijk^	19.49 ± 0.97 ^fghij^	65.61 ± 3.40 ^klm^	88.42 ± 2.61 g^hi^
C-14	256.07 ± 64.92 ^ij^	78.52 ± 5.39 ^cdef^	0.43 ± 0.02 ^jk^	3.90 ± 0.10 ^ghijk^	17.83 ± 0.50 ^hijkl^	31.14 ± 1.75 ^q^	58.84 ± 3.53 ^jkl^
C-17	408.23 ± 18.32 ^fgh^	63.17 ± 5.21 ^ghijkl^	0.37 ± 0.01 ^k^	3.70 ± 0.10 ^jk^	20.09 ± 1.61 ^efghi^	51.67 ± 4.46 ^mno^	89.98 ± 9.56 ^ghi^
C-18	424.34 ± 11.85 ^efg^	71.36 ± 4.47 ^efghij^	0.56 ± 0.04 ^ijk^	4.53 ± 0.06 ^bcd^	24.02 ± 1.46 ^bcd^	58.98 ± 4.61 ^lmn^	123.43 ± 1.01 ^def^
C-27	298.36 ± 13.82 ^ij^	75.64 ± 5.84 ^defgh^	0.75 ± 0.11 ^hij^	5.37 ± 0.15 ^a^	25.22 ± 0.72 ^bc^	33.03 ± 3.16 ^pq^	78.42 ± 2.16 ^hij^
C-28	265.45 ± 35.51 ^ij^	72.92 ± 5.64 ^defghi^	1.05 ± 0.08 ^efgh^	4.73 ± 0.23 ^bc^	29.06 ± 2.35 ^a^	41.02 ± 3.25 ^opq^	100.85 ± 6.99 ^fgh^
C-29	282.34 ± 30.04 ^ij^	79.58 ± 5.50 ^cdef^	0.71 ± 0.39 ^ij^	4.03 ± 0.06 ^efghij^	17.29 ± 1.32 ^hijkl^	49.95 ± 1.05 ^no^	92.56 ± 10.67 ^ghi^
C-30	494.31 ± 33.40 ^def^	55.05 ± 5.49 ^klm^	0.59 ± 0.01 ^ijk^	3.60 ± 0.10 ^k^	18.79 ± 1.19 ^ghijk^	50.38 ± 2.45 ^mno^	56.68 ± 4.57 ^jkl^
C-32	446.48 ± 34.15 ^efg^	38.39 ± 1.11 ^n^	0.55 ± 0.04 ^ijk^	4.23 ± 0.15 ^defgh^	20.22 ± 0.64 ^defgh^	41.70 ± 4.18 ^opq^	60.96 ± 3.00 ^jkl^
C-45	468.09 ± 6.18 ^ef^	61.14 ± 5.85 ^ijklm^	0.65 ± 0.06 ^ijk^	4.00 ± 0.10 ^efghij^	17.19 ± 0.86 ^hijkl^	49.81 ± 4.96 ^no^	61.47 ± 1.15 ^jkl^
C-47	451.30 ± 21.62 ^efg^	91.57 ± 3.76 ^abc^	0.34 ± 0.01 ^k^	3.77 ± 0.06 ^ijk^	19.68 ± 0.77 ^fghij^	47.12 ± 3.10 ^nop^	55.63 ± 5.09 ^kl^
C-48	626.22 ± 36.11 ^b^	64.47 ± 2.07 ^ghijkl^	1.18 ± 0.05 ^def^	4.53 ± 0.15 ^bcd^	23.74 ± 1.10 ^bcde^	150.19 ± 5.94 ^b^	70.19 ± 6.22 ^ijk^
CP-2	349.86 ± 27.48 ^ghi^	67.80 ± 6.81 ^fghijk^	1.36 ± 0.06 ^bcd^	4.30 ± 0.20 ^def^	15.26 ± 0.94 ^klmn^	81.11 ± 6.42 ^ghij^	198.67 ± 13.93 ^b^
CP-3	511.60 ± 49.47 ^cdef^	76.14 ± 2.35 ^defgh^	2.20 ± 0.15 ^a^	4.73 ± 0.12 ^bc^	16.05 ± 1.34 ^jklm^	132.55 ± 5.90 ^c^	237.13 ± 5.14 ^a^
CP-8	300.42 ± 30.41 ^ij^	85.48 ± 2.56 ^abcd^	1.46 ± 0.12 ^bc^	4.20 ± 0.26 ^defgh^	11.51 ± 1.46 ^n^	123.22 ± 2.67 ^cd^	202.94 ± 4.89 ^b^
CP-9	276.46 ± 5.35 ^ij^	71.28 ± 2.22 ^efghij^	1.53 ± 0.03 ^b^	4.30 ± 0.10 ^def^	14.75 ± 1.3 ^lmn^	182.57 ± 8.01 ^a^	158.24 ± 7.41 ^c^
CP-18	284.37 ± 22.95 ^ij^	58.36 ± 3.18 ^jklm^	1.20 ± 0.15 ^bcd^	4.20 ± 0.10 ^defgh^	17.68 ± 0.89 ^hijkl^	124.43 ± 4.32 ^cd^	144.82 ± 10.7 ^cd^
CP-22	272.12 ± 16.28 ^ij^	47.95 ± 1.77 ^mn^	1.50 ± 0.16 ^bc^	3.97 ± 0.12 ^fghijk^	12.75 ± 0.38 ^mn^	113.82 ± 7.22 ^de^	185.12 ± 5.54 ^b^
CP-23	318.35 ± 12.67 ^hij^	53.18 ± 5.42 ^lm^	1.12 ± 0.09 ^efg^	4.27 ± 0.06 ^defg^	15.18 ± 0.93 ^klmn^	115.87 ± 2.32 ^de^	204.91 ± 5.82 ^b^
CP-24	255.73 ± 19.64 ^ij^	80.05 ± 2.45 ^cdef^	1.48 ± 0.09 ^bc^	4.3 ± 0.26 ^def^	12.55 ± 1.32 ^mn^	103.29 ± 6.99 ^ef^	125.64 ± 10.35 ^d^
CP-31	290.10 ± 55.53 ^ij^	82.45 ± 2.65 ^bcde^	1.30 ± 0.04 ^bcd^	4.10 ± 0.10 ^efghi^	16.32 ± 1.00 ^ijklm^	88.37 ± 4.66 ^fgh^	87.60 ± 8.430 ^ghi^
CP-44	224.54 ± 10.60 ^j^	78.70 ± 1.38 ^cdef^	1.29 ± 0.04 ^bcd^	3.87 ± 0.06 ^hijk^	17.32 ± 0.58 ^hijkl^	103.48 ± 9.75 ^ef^	101.83 ± 17.84 ^efg^

Note: Values are expressed as “average concentration ± SD”, and each includes three replicates. Different letters (a, b, c, d, e, f, g, h, I, j, k, l, m, n, o, p, q) indicate statistically significant differences (*p* < 0.05) among columns based on Duncan’s test following the same format.

**Table 2:** Average contents of volatile compounds (n = 3, equivalent of 2-octanol) and their distribution ranges (in parenthesis) of 29 cucumber cultivars.

Code ^a^	Compounds	CAS ^b^	RT (min) ^c^	Average Content (μg/kg FW)
Aldehydes				
A1	Butanal, 3-methyl-	000590-86-3	4.568	6.01 (0–15.7)
A2	Pentanal	000110-62-3	4.563	1.1 (0–9.57)
A3	2-Butenal	004170-30-3	6.08	5.1 (0–15.9)
A4	Hexanal	000066-25-1	7.729	55.50 (32.87–85.95)
A5	2-Pentenal, (E)-	001576-87-0	8.885	0.94 (0–8.72)
A6	2-Pentenal, 2-methyl-	000623-36-9	9.852	2.11 (0–9.53)
A7	Heptanal	000111-71-7	11.047	1.38 (0–6.65)
A8	2-Hexenal, (E)-	006728-26-3	11.804	49.68 (34.32–76.08)
A9	Nonanal	000124-19-6	17.283	60.31 (39.09–142.35)
A10	5-Ethylcyclopent-1-enecarboxaldehyde	036431-60-4	17.566	8.02 (4.13–12.53)
A11	4-Nonenal, (E)-	002277-16-9	18.488	0.44 (0–7.08)
A12	6-Nonenal, (E)-	002277-20-5	18.718	82.20 (0–184.01)
A13	2,4-Heptadienal, (E,E)-	004313-03-5	19.63	60.17 (24.96–91.60)
A14	Benzaldehyde	000100-52-7	20.221	15.99 (0–23.01)
A15	2-Nonenal, (E)-	018829-56-6	20.923	200.70 (93.36–380.42)
A16	2,6-Nonadienal, (E,Z)-	000557-48-2	22.192	302.59 (163.06–479.77)
A17	1-Cyclohexene-1-carboxaldehyde, 2,6,6-trimethyl-	000432-25-7	22.831	11.62 (6.04–22.03)
A18	2,4-Nonadienal, (E,E)-	005910-87-2	24.695	13.54 (0–27.94)
A19	Tridecanal	010486-19-8	27.423	3.96 (1.64–7.61)
A20	4-Oxononanal	1000314-10-4	27.564	4.99 (0–17.03)
A21	Tetradecanal	000124-25-4	29.638	9.04 (3.81–18.59)
A22	Pentadecanal	002765-11-9	31.79	51.99 (18.07–108.02)
A23	cis,cis-7,10,-Hexadecadienal	056829-23-3	35.112	1.29 (0–4.08)
A24	cis-9-Hexadecenal	056219-04-6	36.147	2.51 (0.66–7.01)
Alcohols				
L1	Ethanol	000064-17-5	3.733	3.45 (2.49–6.85)
L2	1-Hexanol	000111-27-3	16.078	13.88 (2.83–32.62)
L3	3-Hexen-1-ol, (E)-	000928-97-2	16.859	1.53 (0–3.24)
L4	1-Hexanol, 2-ethyl-	000104-76-7	19.752	0.48 (0–2.04)
L5	Linalool	000078-70-6	21.221	7.64 (0–18.67)
L6	1-Octanol	000111-87-5	21.518	1.22 (0–3.86)
L7	1-Nonanol	000143-08-8	23.905	15.92 (0–44.2)
L8	3-Nonen-1-ol, (Z)-	010340-23-5	24.407	25.84 (17.55–38.05)
L9	(6Z)-Nonen-1-ol	035854-86-5	25.154	26.48 (8.42–55.49)
L10	3,6-Nonadien-1-ol, (E,Z)-	056805-23-3	25.851	18.01 (0–42.49)
L11	2,6-Nonadien-1-ol	028069-72-9	26.222	0.42 (0–7.65)
L12	Benzyl alcohol	000100-51-6	28.359	0.75 (0–5.19)
L13	11,11-Dimethyl-4,8-dimethylenebicyclo[7.2.0]undecan-3-ol	079580-01-1	36.557	1.03 (0–4.2)
Ketones				
K1	2,3-Pentanedione	000600-14-6	6.807	0.13 (0–1.9)
K2	3-Octanone	000106-68-3	13.063	6.97 (0–12.07)
K3	2-Octanone	000111-13-7	13.98	1.38 (0.79–2.18)
K4	5-Hepten-2-one, 6-methyl-	000110-93-0	15.463	0.7 (0–3.76)
K5	3-Octen-2-one	001669-44-9	17.434	8.21 (0–15.91)
K6	3,5-Octadien-2-one	038284-27-4	20.352	52.86 (37.61–72.36)
K7	3,5-Octadien-2-one, (E,E)-	030086-02-3	21.592	18.29 (0–46.01)
K8	.alpha.-Ionone	000127-41-3	28.018	1.1 (0–2.92)
K9	5,9-Undecadien-2-one, 6,10-dimethyl-, (E)-	003796-70-1	28.125	0.07 (0–0.76)
K10	trans-.beta.-Ionone	000079-77-6	29.838	11.25 (5.25–19.65)
K11	3-Buten-2-one, 4-(2,2,6-trimethyl-7-oxabicyclo[4.1.0]hept-1-yl)-	023267-57-4	30.877	1.83 (0–3.83)
K12	2(3H)-Furanone, dihydro-5-pentyl-	000104-61-0	31.492	0.15 (0–1.68)
Alkenes				
O1	1-Octene	000111-66-0	2.64	1.61 (0–2.8)
O2	Decane	000124-18-5	5.28	0.13 (0–2.79)
O3	(1R)-2,6,6-Trimethylbicyclo[3.1.1]hept-2-ene	007785-70-8	5.651	0.96 (0–15.84)
O4	Dodecane	000112-40-3	11.633	0.1 (0–1.42)
O5	Tridecane	000629-50-5	14.819	0.41 (0–2.51)
O6	1,6-Dioxaspiro[4.4]nonane, 2-ethyl-	038401-84-2	15.985	0.08 (0–1.03)
O7	Tetradecane	000629-59-4	17.737	0.3 (0–3.54)
O8	Caryophyllene	000087-44-5	22.446	6.03 (0–28.34)
O9	Humulene	006753-98-6	24.051	9.63 (0–26.8)
O10	Nonadecane	000629-92-5	29.35	1.14 (0–2.12)
O11	Caryophyllene oxide	001139-30-6	30.755	5.39 (0–19.7)
O12	(+)-epi-Bicyclosesquiphellandrene	054274-73-6	34.332	0.33 (0–1.59)
Others				
E1	2(4H)-Benzofuranone, 5,6,7,7a-tetrahydro-4,4,7a-trimethyl-	015356-74-8	37.22	1.07 (0–6.22)
E2	Furan, 2-ethyl-	003208-16-0	3.997	7.81 (2.23–16.33)
E3	Furan, 2-pentyl-	003777-69-3	12.306	15.38 (2.06–41.29)
E4	cis-2-(2-Pentenyl)furan	070424-13-4	14.409	10.36 (0–29.52)
E5	Furan, 2-(2-propenyl)-	075135-41-0	22.636	1.3 (0–2.59)
E6	Furan, 2-(1-pentenyl)-, (E)-	036144-40-8	28.769	3.13 (0–5.3)

^a^ Compound codes, ^b^ CAS number, ^c^ Retention time (min).

**Table 3:** Number of volatile compounds and total content of volatiles identified in the 29 cucumber cultivars.

NO	Cultivars	Number of Volatile Compounds	Total Content (μg/kg FW)
1	CP-3	45	1187.8 ± 133.89 ^efgh^
2	CP-8	44	1047.63 ± 56.3 ^hi^
3	CP-9	46	916.15 ± 73.22 ^ij^
4	CP-22	42	1146.39 ± 37.35 ^fgh^
5	CP-23	43	1116.71 ± 104.9 ^gh^
6	C-1	45	1167.1 ± 74.95 ^efgh^
7	C-2	43	1236.5 ± 51.64 ^defg^
8	C-3	46	1121.65 ± 75.33 ^gh^
9	C-4	38	1332.14 ± 28.61 ^cde^
10	C-6	40	1142.65 ± 50.04 ^fgh^
11	C-9	45	1802.38 ± 53.11 ^a^
12	C-11	47	1390.91 ± 42.11 ^cd^
13	C-13	44	1172.3 ± 18.71 ^efgh^
14	C-14	38	1564.49 ± 112.04 ^b^
15	C-17	48	1473.07 ± 155.47 ^bc^
16	C-18	47	1571.32 ± 78.57 ^b^
17	C-27	50	1296.37 ± 97.21 ^def^
18	C-28	45	1630.5 ± 188.25 ^b^
19	C-29	45	1086.09 ± 71.26 ^gh^
20	C-30	46	1042.61 ± 107.14 ^hi^
21	C-32	49	1294.13 ± 98.03 ^def^
22	C-45	48	1037.35 ± 105.59 ^hi^
23	C-47	45	1562.69 ± 81.07 ^b^
24	C-48	49	1099.04 ± 126.96 ^gh^
25	CP-2	40	1076.46 ± 111.91 ^ghi^
26	CP-18	47	1374.38 ± 61.57 ^cd^
27	CP-24	47	1059.67 ± 20.6 ^hi^
28	CP-31	45	771.34 ± 66.75 ^j^
29	CP-44	51	1125.12 ± 76.06 ^fgh^

Note: Values are expressed as “average concentration ± SD”, and each includes three replicates. Different letters (a, b, c, d, e, f, g, h, i, j) indicate statistically significant differences (*p* < 0.05) among columns based on Duncan’s test.

**Table 4:** Total content (μg/kg FW) of each type of volatiles in 29 cucumber cultivars.

Cultivars	Aldehydes	Alcohols	Ketones	Alkenes	Others
C-1	895.1 ± 54.65 ^hijkl^	80.14 ± 10.55 ^kl^	113.30 ± 12.88 ^defg^	49.41 ± 1.60 ^b^	29.17 ± 3.45 ^efgh^
C-2	1002.69 ± 41.44 ^gfh^	70.35 ± 1.64 ^l^	118.88 ± 5.63 ^cde^	3.93 ± 0.57 ^ij^	40.65 ± 4.35 ^bcde^
C-3	860.42 ± 57.35 ^ijklm^	78.76 ± 4.89 ^kl^	98.15 ± 8.86 ^gh^	37.00 ± 1.52 ^cd^	47.33 ± 3.10 ^bc^
C-4	1097.62 ± 38.57 ^def^	86.13 ± 2.46 ^jkl^	99.72 ± 3.38 ^fgh^	20.53 ± 21.02 ^ef^	28.16 ± 9.2 ^efghi^
C-6	980.68 ± 50.43 ^fghi^	73.18 ± 0.70 ^kl^	72.89 ± 2.87 ^jk^	0.83 ± 0.07 ^j^	15.08 ± 0.99 ^ghi^
C-9	1531.18 ± 36.56 ^a^	99.48 ± 7.18 ^hij^	104.92 ± 6.8 ^efgh^	32.76 ± 2.29 ^d^	34.06 ± 1.59 ^cdef^
C-11	1094.46 ± 39.67 ^def^	90.62 ± 5.64 ^ijk^	125.65 ± 3.73 ^bcd^	46.45 ± 0.49 ^b^	33.74 ± 0.50 ^cdef^
C-13	966.76 ± 16.87 ^ghij^	68.68 ± 3.70 ^l^	108.40 ± 2.74 ^efgh^	3.49 ± 0.12 ^ij^	24.98 ± 1.61 ^efghi^
C-14	1312.05 ± 95.82 ^b^	118.93 ± 2.55 ^fgh^	66.81 ± 1.79 ^kl^	23.39 ± 0.95 ^e^	43.32 ± 13.43 ^bcd^
C-17	1143.73 ± 113.81 ^cde^	71.69 ± 3.12 ^l^	139.37 ± 7.73 ^ab^	43.76 ± 2.56 ^bc^	74.52 ± 30.13 ^a^
C-18	1224.38 ± 60.35 ^bc^	85.43 ± 6.44 ^jkl^	134.24 ± 6.55 ^ab^	73.59 ± 4.37 ^a^	53.68 ± 4.45 ^b^
CP-3	955.40 ± 108.83 ^ghijk^	111.63 ± 10.12 ^fgh^	77.38 ± 10.12 ^jk^	23.12 ± 2.78 ^e^	20.25 ± 2.65 ^fghi^
CP-8	828.83 ± 45.95 ^klmno^	119.53 ± 9.39 ^fgh^	81.21 ± 0.85 ^ijk^	9.95 ± 0.28 ^ghi^	8.13 ± 0.1 ^i^
CP-9	701.32 ± 58.68 ^p^	108.67 ± 5.28 ^gh^	78.15 ± 9.06 ^jk^	12.52 ± 0.71 ^fgh^	15.47 ± 1.08 ^ghi^
CP-22	886.13 ± 29.44 ^hijklm^	122.15 ± 4.57 ^fgh^	114.17 ± 7.22 ^def^	12.79 ± 0.49 ^fgh^	11.14 ± 4.18 ^hi^
CP-23	892.26 ± 88.23 ^hijkl^	97.87 ± 5.97 ^hij^	103.91 ± 9.10 ^efgh^	7.66 ± 0.90 ^hij^	15.00 ± 1.26 ^ghi^
C-27	925.38 ± 60.8 ^ghijk^	104.86 ± 6.29 ^ghi^	139.89 ± 8.48 ^ab^	36.29 ± 3.07 ^cd^	89.95 ± 22.21 ^a^
C-28	1201.66 ± 136.22 ^bcd^	179.85 ± 21.47 ^bc^	131.08 ± 15.93 ^abc^	36.77 ± 3.74 ^cd^	81.15 ± 11.37 ^a^
C-29	782.44 ± 60.44 ^lmnop^	112.17 ± 3.86 ^fgh^	104.69 ± 8.91 ^efgh^	33.51 ± 1.83 ^d^	53.27 ± 3.81 ^b^
C-30	711.16 ± 73.91 ^op^	190.02 ± 14.81 ^ab^	84.07 ± 9.19 ^ij^	23.14 ± 2.51 ^e^	34.22 ± 7.59 ^cdef^
C-32	850.30 ± 62.87 ^jklmn^	202.17 ± 16.08 ^a^	141.72 ± 9.64 ^a^	16.85 ± 1.51 ^efg^	83.09 ± 7.96 ^a^
C-45	745.79 ± 68.30 ^nop^	144.67 ± 16.58 ^e^	77.42 ± 7.76 ^jk^	24.14 ± 4.39 ^e^	45.32 ± 9.20 ^bcd^
C-47	1188.44 ± 55.88 ^cd^	105.51 ± 5.34 ^ghi^	119.72 ± 6.72 ^cde^	70.88 ± 5.73 ^a^	78.15 ± 7.50 ^a^
C-48	780.50 ± 84.15 ^lmnop^	131.27 ± 7.32 ^ef^	95.14 ± 17.85 ^hi^	43.42 ± 4.72 ^bc^	48.73 ± 14.68 ^bc^
CP-2	886.40 ± 76.29 ^hijklm^	104.18 ± 23.57 ^ghi^	56.86 ± 5.22 ^l^	9.10 ± 2.59 ^ghij^	19.92 ± 6.38 ^fghi^
CP-18	1041.32 ± 43.66 ^efg^	162.39 ± 10.68 ^d^	104.52 ± 5.24 ^efgh^	14.30 ± 0.76 ^fgh^	51.85 ± 2.61 ^b^
CP-24	759.19 ± 22.22 ^mnop^	165.94 ± 1.46 ^cd^	112.88 ± 1.90 ^defg^	11.83 ± 0.41 ^ghi^	9.82 ± 1.29 ^i^
CP-31	548.39 ± 52.23 ^q^	128.54 ± 4.54 ^efg^	75.36 ± 6.67 ^jk^	10.44 ± 0.78 ^ghi^	8.62 ± 2.86 ^i^
CP-44	790.52 ± 54.65 ^lmnop^	170.87 ± 8.07 ^cd^	105.24 ± 6.26 ^efgh^	24.87 ± 1.51 ^e^	33.61 ± 5.69 ^cdef^

Note: Values are expressed as “average concentration ± SD”, and each includes three replicates. Different letters (a, b, c, d, e, f, g, h, I, j, k, l, m, n, o, p, q) indicate statistically significant differences (*p* < 0.05) among columns based on Duncan’s test.

## Data Availability

The datasets generated for this study are available on request to the corresponding author.

## Supplementary Materials

The following supporting information can be downloaded at: https://www.mdpi.com/article/10.3390/foods14223878/s1, Table S1. Cucumber cultivars used in this study and some basic fruit quality parameters. Table S2. The content (µg/kg FW) of 21 common volatile compounds in 29 cucumber cultivars. Table S3. Relative contents (µg/kg FW) of identified volatiles in the fruits of 29 cucumber cultivars. Table S4. Percentage (%) of each type of volatiles in 29 cucumber cultivars. Table S5. VIP value for sample line. Table S6. VIP value for sample groups of types.
